# Personalized robotic interventions for apathy in dementia: the RAPHAel trial

**DOI:** 10.3389/frobt.2026.1913791

**Published:** 2026-09-08

**Authors:** Claudia Casadio, Vanessa Zanelli, Valentina Bettelli, Francesco Ricci, Lucrezia Grassi, Pietro Benvenuti, Erika Rovini, Giulia Smecca, Sara Isernia, Simone Salemme, Manuela Tondelli, Annalisa Chiari, Francesca Baglio, Filippo Cavallo, Fausta Lui, Francesca Benuzzi, Laura Fiorini, Antonio Sgorbissa, Giovanna Zamboni

**Affiliations:** 1 Department of Biomedical, Metabolic and Neural Sciences, University of Modena and Reggio Emilia, Modena, Italy; 2 Department of Computer Science, Bioengineering, Robotics and Systems Engineering University of Genoa, Genoa, Italy; 3 Department of Industrial Engineering, University of Florence, Florence, Italy; 4 Fondazione Don Carlo Gnocchi ETS of Milan, Milan, Italy; 5 University Hospital of Modena, Modena, Italy

**Keywords:** apathy, dementia, mild cognitive impairment, MCI, robotic intervention, telerehabilitation

## Abstract

**Background:**

Apathy is common in individuals with mild cognitive impairment (MCI) or dementia and mild behavioral impairment (MBI), and is associated with reduced quality of life and increased caregiver burden. Home-based robot-assisted interventions offer a novel approach for cognitive stimulation and behavioral management, but robust evidence on feasibility, acceptability, and clinical impact is limited.

**Objectives:**

To evaluate the effects of home-based robotic interventions on the level of apathy in individuals with cognitive decline, including MCI, MBI, and dementia. Secondary objectives are to assess the usability of the robotic devices and the extent of patients’ interactions with and through these technologies, as well as to evaluate the effects of the robotic interventions on caregivers’ stress levels and quality of life.

**Methods:**

This single-blind, randomized controlled trial includes three arms: two experimental robot-assisted interventions (tele-operated cognitive stimulation and humanoid robot) and an active control group receiving occupational therapy, which represents the current gold-standard intervention. Eighty patients with MCI, MBI, or dementia, and clinically significant apathy, together with their study partners, will be recruited from neurology, geriatrics, and neurorehabilitation centers in Italy. Each intervention will last 6 weeks. Assessments will be performed at baseline (T0), post-intervention (T1), and follow-up (T2) and will include clinical questionnaires, cognitive and social cognition tasks, and sensor-based measures of social grasping.

**Conclusion:**

This study will provide evidence on the potential clinical benefits, feasibility, and acceptability of home-based robot-assisted interventions for apathy in cognitive decline, as well as inform the development of digital markers for personalized monitoring and intervention.

## 1 Introduction

In Western countries, and specifically in Italy, the prevalence and socio-economic impact of dementia, or major neurocognitive disorder, are highly significant, partly due to the progressive aging of the population. Indeed, the proportion of individuals aged over 65 in Italy has exceeded 23% of the total population (ISTAT, 2020), and the number of individuals living with dementia is now estimated at over 600,000. In addition, the domestic care sector in Italy has reached nearly €17 billion, with the annual cost for a regularly employed live-in caregiver estimated at approximately €20,270 (CENSIS, 2024). This burden remains profoundly privatized, as 64.3% of families act as primary caregivers, leading to significant repercussions, including pervasive psychological stress for 88.3% of individuals and substantial professional limitations for 93.4% of the women involved (CENSIS, 2024).

From a clinical perspective, the dementia stage represents the final phase of a process that is often preceded by milder forms of cognitive decline, which fall along a continuum between normal cognitive functioning and dementia. These conditions are variously referred to as Mild Cognitive Impairment (MCI; Winblad et al., 2004) or minor neurocognitive disorder (American Psychiatric Association, 2013). MCI is characterized by a slight degree of cognitive deterioration that, although reported by the individual and/or family members and objectively detectable through psychometric testing, is not sufficiently severe to significantly interfere with daily functioning (Winblad et al., 2004).

Alongside MCI, which focuses on the presence of cognitive symptoms, the diagnostic construct of Mild Behavioral Impairment (MBI; Taragano et al., 2008) has also been introduced. In contrast to MCI, MBI identifies a condition characterized by the presence of mild neuropsychiatric symptoms even in the absence of significant cognitive decline. This construct may help identify individuals at risk of developing dementia, highlighting the importance of recognizing and addressing not only cognitive decline, but also behavioral changes (Ismail et al., 2015).

Behavioral and psychological symptoms of dementia (BPSD; Finkel et al., 1996) associated with neurodegenerative diseases may emerge at very early stages and can represent a distinguishing feature of certain atypical dementias, such as frontotemporal dementia or the dysexecutive/frontal variant of Alzheimer’s disease. Apathy, anhedonia, and lack of interest are among the most frequent BPSD and are often perceived as particularly burdensome in the relationship between the individual and the caregiver (Mortby et al., 2022). However, to date, no specific pharmacological intervention has proven effective in the management of apathy (NICE, 2018). Some evidence in the literature supports the effectiveness of non-pharmacological interventions for the treatment of non-cognitive symptoms in individuals with dementia or MCI; however, only a limited number of studies have specifically used improvement in apathy as an outcome measure (NICE, 2018; Koh et al., 1994; Oliveira et al., 2021; O'Connor et al., 2019). Some studies highlighted the role of occupational therapy (OT) in improving BPSD through personalized, home-based activity programs designed to engage individuals with dementia according to their abilities and living context. These interventions often involve caregiver participation and environmental adaptation within each patient’s home. Most OT interventions demonstrated benefits in enhancing social participation, overall wellbeing, and certain BPSD, such as depression and agitation (Graff et al., 2008; Gitlin et al., 2010), although they generally do not show a specific effect on apathy, with few exceptions (Nishida et al., 2017).

Furthermore, few studies examined the effects of robotic interventions on apathy in individuals with dementia or MCI. Existing studies primarily focused on assistive rather than rehabilitative purposes, and yielded limited evidence of effectiveness in improving apathy (Valentí Soler et al., 2015; Mancioppi et al., 2019; Yu et al., 2022).

Therefore, this paper presents the study protocol for the RAPHAel project, which aims to evaluate the efficacy of non-pharmacological interventions targeting apathy in individuals with dementia, MCI, or MBI. The study compares two robot-assisted intervention strategies, a tele-operated cognitive stimulation program and an autonomous conversational robot, against an active occupational therapy control condition, which is currently considered the gold standard.

## 2 Materials and methods

The study adheres to the SPIRIT (Standard Protocol Items: Recommendations for Interventional Trials) guidelines and will be conducted in accordance with the principles of the Declaration of Helsinki (World Medical Association, 2013), Good Clinical Practice (GCP), in compliance with Italian law. The study has received ethical approval (Comitato Etico Area Vasta Emilia Nord; protocol number: 263/2024/DISP/AOUMO) and is registered in clinicaltrials.gov (NCT07404410). All patients and their study partners must give their written informed consent before participating in the study.

### 2.1 Participants

In each recruiting center (University Hospital of Modena and Fondazione Don Carlo Gnocchi of Milan), a neurologist or geriatrician will identify patients with MCI, MBI, or dementia exhibiting clinically significant apathy. Eligible patients will be informed about the study by the physician, provided with the participant information sheet, and invited to consider participation together with a study partner (who could be a family member, friend, or a formal caregiver). If both patient and study partner express interest and meet the eligibility criteria, a study researcher will contact them to provide detailed information and to answer any questions regarding the study.

Inclusion Criteria for Patient:

- Age >40 years;
- Clinical diagnosis of mild or major neurocognitive disorder due to Alzheimer’s disease, frontotemporal dementia, Lewy body disease, or another neurodegenerative condition associated with apathy, as documented by a Neuropsychiatric Inventory (NPI) sub-item G–Apathy score >2;
- Sufficient occupational history to exclude intellectual disability;
- Preserved abilities in comprehension and production of written and spoken language;
- Absence of agitation or aggression, as documented by an NPI sub-item C–Agitation/Aggression score <2;
- Availability of a study partner (family member, friend, or caregiver) who knows the participant well, is willing to respond to questionnaires regarding the participant, and is able to read, understand, and speak Italian to provide independent consent for their own participation.

Exclusion Criteria for Patient:

- Failure or withdrawal of consent;
- History or presence of central nervous system disorders that could impact cognitive functions other than those under study (e.g., severe cerebrovascular accidents, brain tumors, normal pressure hydrocephalus, traumatic brain injury, etc.);
- History or presence of significant psychiatric disorders;
- Presence of medical conditions potentially interfering with cognitive functions (e.g., renal or hepatic failure, obstructive sleep apnea, hypothyroidism, vitamin B12 deficiency, etc.).

Inclusion Criteria for Study Partner:

- Family member, friend, or caregiver of the primary participant;
- Interaction with the participant at least once a week, for at least 10 h per week (or more);
- Willingness to respond to questions and questionnaires regarding the participant’s lifestyle, behavior, and symptoms;
- Ability to read, understand, and speak Italian.

Exclusion Criteria for Study Partner:

- Failure or withdrawal of consent to participate as a study partner;
- Failure or withdrawal of consent by the primary participant.

### 2.2 Experimental procedure

**FIGURE 1:**
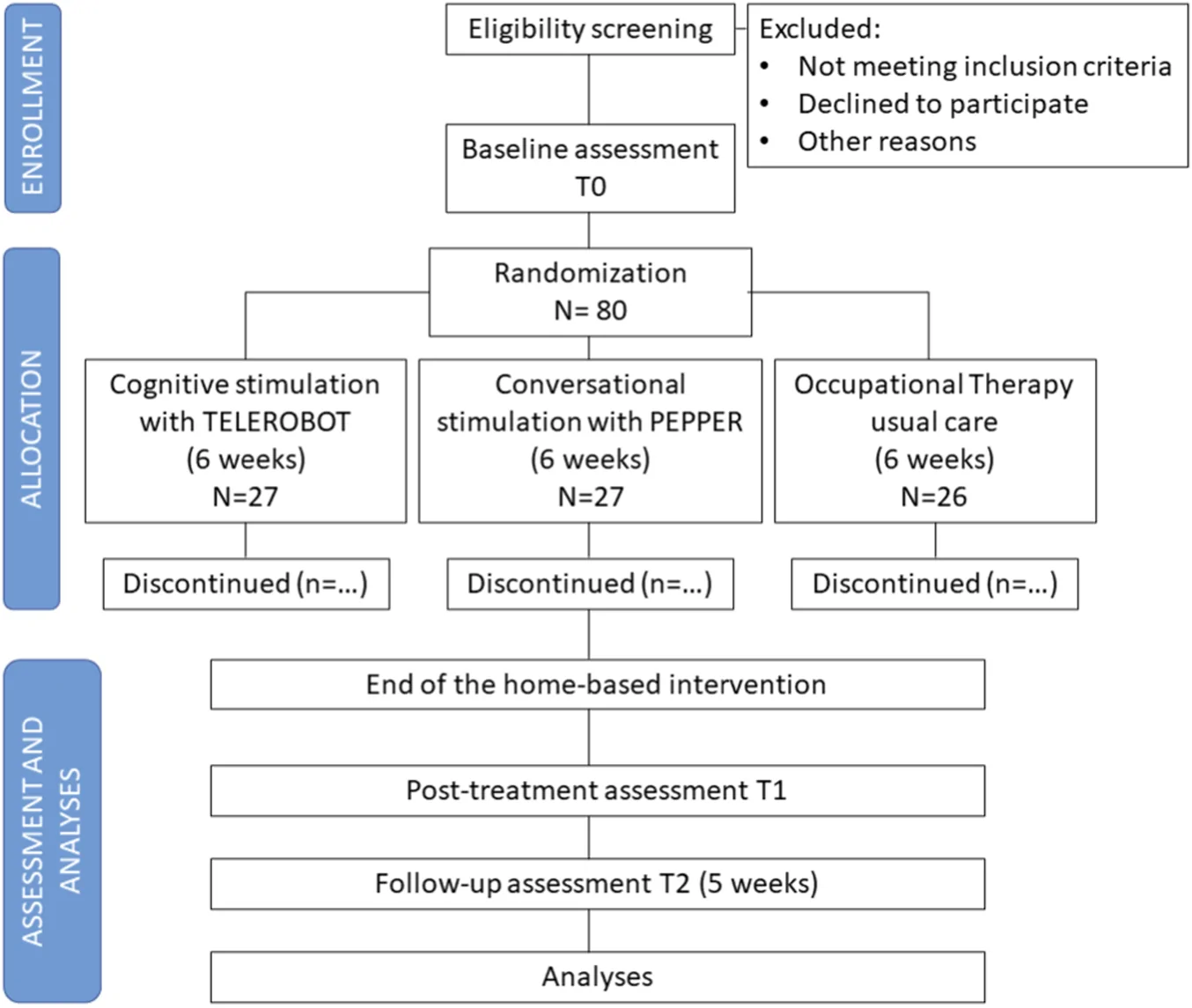
Trial work plan.

Each participant will be assigned a unique identification code at the beginning of the study to ensure anonymity. The study follows a structured sequence of baseline assessment (T0), intervention, post-intervention assessment (T1), and follow-up (T2), as illustrated in Figure 1. All outcome assessments will be conducted by blinded evaluators, who will remain unaware of the patients’ group allocation.

#### 2.2.1 Assessment

At baseline (T0), post-intervention (T1, 6 weeks after T0), and follow-up assessment (T2, 5 weeks after T1), patients and their study partners will complete a comprehensive set of clinical, cognitive, behavioral, and psychosocial assessments, including:

- Neuropsychiatric Inventory (NPI; Cummings et al., 1994; Italian version by Binetti et al., 1998): assessment of non-cognitive behavioral disturbances in dementia across multiple domains, including frequency, severity, and caregiver distress.
- Apathy Evaluation Scale (AES; Marin et al., 1991; Italian version by Borgi et al., 2016): measurement of apathy in behavioral, cognitive, and emotional domains.
- Yoni-48 Task (Italian version by Isernia et al., 2022b; Isernia et al., 2023): computerized evaluation of first- and second-order affective and cognitive Theory of Mind (ToM).
- Edinburgh Social Cognition Test (ESCoT; Italian version by Isernia et al., 2022a): assessment of cognitive and affective ToM and understanding of interpersonal and intrapersonal social norms.
- International Affective Picture System (IAPS; Lang et al., 1997) with Self-Assessment Manikin (SAM; Bradley and Lang, 1994): evaluation of emotional valence and physiological arousal.
- Zarit Burden Inventory (Zarit et al., 1980; Italian version by Chattat et al., 2011): caregiver burden assessment.
- Quality of Life in Alzheimer’s Disease (QoL-AD; Logsdon et al., 1999; Italian version by Bianchetti et al., 2017): multidimensional quality-of-life assessment combining patient and caregiver ratings.
- Cognitive Reserve Index Questionnaire (CRIq; Italian version by Nucci et al., 2012): evaluation of cognitive reserve across education, occupational, and leisure activities.
- Cognitive assessments, including the Montreal Cognitive Assessment (MoCA; Nasreddine et al., 2005; Italian version by Santangelo et al., 2015).

The usability and the perceived usefulness, ease of use, and overall acceptance of the robotic systems will be subjectively evaluated at T1 using the System Usability Scale (SUS) and the Technology Acceptance Model (TAM).

During T0, T1 and T2, patients will also perform a grasping task using the BMR4RAPHAEL system (Rovini et al., 2021), which includes the SensHand device, a wearable 6-axis inertial measurement unit (IMU) with a sampling frequency of 50 Hz, and a custom graphical data acquisition interface. Patients will sit in front of a table with their right hand in a resting position. A cylindrical object will be positioned in front of the participant along the midsagittal axis. Patients will complete:

1. Individual condition: reaching, grasping, and moving an object between designated supports.
2. Social condition: reaching, grasping, and handing an object to another person seated at the table, with matched target location across conditions.

#### 2.2.2 Rehabilitation interventions

Following baseline assessment, patients will be randomized (1:1:1) to one of three intervention arms; all the interventions will be carried out with patients at their own homes, and will last 6 weeks:

1. Tele-operated robot-mediated intervention: patients will undergo a remotely delivered cognitive stimulation program, via the tele-operated robot RoboXcare (SCAI SOLUTION Group S.p.A). Sessions will be led by a qualified neuropsychologist three times per week; each will last around 45 min, and will be scheduled to integrate into the patient’s daily routine. The intervention will employ previously validated cognitive stimulation protocols (Realdon et al., 2016; Rossetto et al., 2023) designed to target multiple domains, including attention, reasoning, executive functions, and visual-spatial skills, alongside procedural, semantic, and autobiographical memory. To ensure progressive stimulation, the difficulty level of these activities will be increased at fixed weekly intervals.
2. Conversational robot-mediated intervention: patients will engage in personalized verbal and non-verbal interactions with the humanoid robot Pepper (SoftBank Robotics), connected to the CAIR (Culture-Aware AI and Robotics) system, a cloud-based architecture for diversity-aware and situated human–robot interaction previously presented and progressively extended in prior work (Grassi, 2024; Grassi et al., 2025). This cloud-based system supports tailored dialogues through a dedicated knowledge base and enables the integration of participant-specific information, including cultural background, language preferences, individual interests, and social relationships, entered via a tablet interface. The robotic system will be programmed to proactively initiate social interactions at least three times per week, to promote social engagement and reduce social withdrawal. Patients will also be allowed to initiate unlimited additional sessions independently; the frequency and duration of all interactions will be automatically recorded. To support sustained engagement, the CAIR server will systematically track interaction metrics and use them to adapt future conversations according to the topics and interaction patterns associated with higher individual engagement.
3. Human-based intervention: patients will undergo a structured occupational therapy program focused on the prevention and management of behavioral and psychological symptoms of dementia (BPSD). This intervention will be delivered by a qualified occupational therapist in accordance with the Essential Levels of Assistance (Ministero della Salute, DPCM, 2017) and will include specialized caregiver training alongside home environment adaptation. The program will provide eight home-based sessions and two telephone-based sessions, specifically designed to enhance the quality of life and reduce the psychological burden for both the participant and their study partner. These personalized activities are tailored to the patient’s functional abilities and domestic context to promote social participation and emotional wellbeing.

For robot-mediated interventions, an occupational therapist will install the device at the patient’s home and provide a 4G connection if needed. Patients and study partners will be instructed on device operation.

It could be argued that across the three intervention arms the substantial difference in contact time represents a limitation of the study protocol. However, this difference is an intrinsic consequence of the distinct nature of each intervention: the control arm adheres to the frequency defined by national care indications and therefore represents the best possible standard of care (Ministero della Salute, DPCM, 2017), the tele-operated robot follows a fixed schedule of three 45-min sessions/week, consistent with previous protocols (Realdon et al., 2016; Rossetto et al., 2023), and the Pepper robot, being autonomously available at the patient’s home, allows potentially unrestricted, self-paced interaction. Nonetheless, all these interventions pursue the same clinical objective—the reduction of apathy in patients with dementia. Hence, this heterogeneity in contact time is acknowledged as a limitation, thus it will be taken into account in the statistical analyses and in the interpretation of the results.

### 2.3 Trial design

The study is designed as a single-blind, randomized controlled trial (RCT). Patients with neurocognitive disorders (both minor - i.e., MCI - and major, i.e., dementia), and presenting either MBI or BPSD, will be recruited from the Complex Units of Neurology and Geriatrics of the Azienda Ospedaliero-Universitaria di Modena (Modena), as well as from the Neurorehabilitation Units of the IRCCS Fondazione Don Carlo Gnocchi (Milan). After recruitment, individuals will be randomly assigned to one of two experimental groups involving robotic interventions (i.e., Telepresence robot or Pepper Robot) or to the active control group. Both the robotic and occupational therapy interventions will be conducted over 6 weeks. Primary and secondary outcome measures will be collected at baseline (T0), immediately post-intervention (T1, 6 weeks), and at follow-up (T2, 5 weeks after the end of the intervention, i.e., 11 weeks total time). The trial work plan is illustrated in Figure 1.

### 2.4 Randomization and blinding

Eligible patients will be randomized using the “blockrand” package in (version 4.1.2; R Core Team, 2021). Randomization will be performed separately for each recruitment center and, within each center, will be stratified according to clinical and demographics variables, including diagnosis, sex, age, years of education, MOntreal Cognitive Assessment (MOCA) score, and apathy severity as measured by the Neuropsychiatric Inventory (NPI) apathy subscale. The allocation sequence will be prepared by a researcher not involved in either treatment delivery or outcome assessments. Investigators responsible for treatment administration and evaluations will not have access to the randomization list. Once generated, the list will be uploaded to REDCap in a non-visible format. Upon the creation of each new patient record and study ID, REDCap will automatically assign patients to their study group according to the predefined allocation sequence. In addition, different user access levels within REDCap will ensure that group allocation remains concealed from outcome assessors.

Patients will be randomly assigned to one of the two experimental groups or to the active control group (1:1:1 ratio).

Given the nature of the interventions, blinding will not be feasible for recruiting clinicians, researchers administering the interventions, or patients. However, outcome assessments at baseline (T0), post-intervention (T1), and follow-up (T2), including clinical endpoints and clinical/psychological questionnaires, will be conducted by trained evaluators who are blinded to group allocation. In addition, data analysts will remain blinded to treatment assignment throughout the analysis phase.

## 3 Outcome measures

### 3.1 Primary outcome measures

The primary objective of this study is assessing the clinical and behavioral effects of home-based robot interventions compared to standard occupational therapy.

The primary endpoints of the study are defined as follows:

1. Clinical improvement of apathy in patients, assessed using:
  - Apathy Evaluation Scale (AES)
  - Yoni-48 Task
  - Edinburgh Social Cognition Test (ESCoT)
  - International Affective Picture System (IAPS)
2. Interaction metrics with robotic systems, including:
  - Response times of system components
  - Number and type of conversation topics addressed, and number of characters in each sentence, spoken per topic (e.g., sports, cinema, cooking, travel)
  - Tone of verbal responses (neutral, humorous, aggressive, etc.)
  - Attitude toward discussion topics (positive, negative, neutral)
  - Objects recognized in the environment, along with labels that classify the identified items and their spatial relationships—for example, a lamp or a vase placed on a table.
  - Number of verbal interactions per minute with the robot
  - Number of physical interactions per minute (e.g., hand, head, tablet, or base contact with the robot).

These interaction metrics, namely, the frequency and spontaneity of verbal and physical engagement with the Pepper robot, will also be interpreted as clinical indicators of the patient’s willingness to engage, given that reduced initiation and engagement are core features of apathy.

3. Sensor-derived and kinematic measures during social grasping tasks, collected via BMR4RAPHAEL wearable sensors and cameras:
  - Integral of the acceleration vector (m/s^2^)
  - Peak velocity amplitude (m/s) and timing
  - Execution time, reaction time, and deceleration (absolute and relative)
  - Maximum hand excursion (degrees) and timing
  - Asymmetry and kurtosis of movement.

### 3.2 Secondary outcome measures

Secondary objectives are to evaluate the acceptability and feasibility of home-based robot-assisted interventions in individuals with cognitive or behavioral impairment exhibiting apathy, as well as the impact on caregiver burden and quality of life.

Secondary endpoints include:

1. Participant and caregiver completion of the System Usability Scale (SUS; Brooke, 1996) to assess perceived usability of the robotic devices;
2. Participant and caregiver responses on the Technology Acceptance Model (TAM; Venkatesh and Bala, 2008) questionnaire, evaluating perceived usefulness, ease of use, and overall acceptance of the technology.
3. Caregiver outcomes, including stress levels and quality of life, assessed using:
  - Neuropsychiatric Inventory (NPI)
  - Zarit Burden Inventory (ZBI)
  - Quality of Life in Alzheimer’s Disease (QoL-AD).

### 3.3 Data collection

All study data will be pseudonymized to protect participant privacy. Original, identifiable data will be securely stored by each participating center in compliance with current Italian law on the protection and processing of personal and sensitive data, and the EU General Data Protection Regulation (GDPR 2016/679).

To maintain methodological rigor, a single blinded neuropsychologist will conduct all primary and secondary outcome evaluations for each participant throughout the study (T0, T1, and T2). Clinical, behavioral, and caregiver-reported data, as well as device-derived and sensor-based measurements, will be collected according to standardized procedures at each data collection point (T0, T1, T2).

We are committed to the highest standards of data integrity; all clinical, functional, and neurocognitive data will be collected by specialists and housed in a secure, dedicated dataset. Furthermore, the storage and processing of all research data will be performed in full compliance with established best practices and only after obtaining informed consent from each participant. All data will be entered in digital format, with quality control checks performed to ensure accuracy, completeness, and consistency across centers. Each recruiting center, as the autonomous data manager for the data collected at its site or through its devices, will have access to pseudonymized datasets stored on the REDCap platform at Fondazione Don Carlo Gnocchi of Milan, in accordance with the shared study protocol. Patients’ unique identification codes will be used consistently across all data collection points (T0, T1, T2) to ensure anonymity while allowing longitudinal tracking of outcomes.

### 3.4 Statistical analysis

Initial data inspection will include descriptive statistics to summarize demographic and clinical characteristics and to identify potential outliers. Outliers will be evaluated according to standard practices, including visual inspection of boxplots and standardized residuals (values exceeding ±3 standard deviations), and decisions on their treatment (e.g., retention, transformation, or exclusion) will be justified and documented.

The distribution of each variable will be assessed using both visual (histograms, Q-Q plots) and statistical methods (Shapiro-Wilk test) to guide the choice of parametric or non-parametric analyses. For the main analyses, a two-way repeated-measures ANOVA will be conducted with Group (three levels: two robot-assisted interventions and one active control) and Time (three levels: T0, T1, T2) as factors. Post-hoc comparisons will be performed with appropriate correction for multiple testing.

Additionally, correlation analyses and regression models (both linear and logistic, where appropriate) will be conducted to explore associations between clinical outcomes, caregiver measures, and device-derived metrics, as well as potential predictive factors. All analyses will be conducted using (version 4.1.2; R Core Team, 2021) and Matlab (The Math Works, Inc., 2025) and/or equivalent statistical software, and significance thresholds will be set *a priori*.

Results will be reported as aggregated data to ensure participant anonymity.

Clinical data will be analyzed at the Department of Biomedical, Metabolic, and Neuroscience of the University of Modena and Reggio Emilia.

Device- and sensor-derived data, including kinematic and interaction metrics from robot-assisted interventions, will be analyzed in relation to clinical and behavioral outcomes by experts at the University of Genoa and the University of Florence.

### 3.5 Sample size calculation

To the best of our knowledge, robust evidence on the effect size of robot-mediated interventions primarily aimed at usability and behavioral outcomes in individuals with neurocognitive disorders is currently lacking. Therefore, a formal sample size calculation based on statistical power is not feasible. The proposed sample size is determined according to a pragmatic approach, taking into account the epidemiology of apathy within the target population and previous studies in the field of robotic interventions and dementia, which typically include small sample sizes (often fewer than 20 patients per arm; Hung et al., 2021; Mancioppi et al., 2019; Moyle et al., 2019).

In line with these considerations, the study aims to enroll approximately 25 patients per arm who will complete the trial. Accounting for an expected attrition rate of approximately 6%, the total sample to be randomized was set at 80 patients, allocated with a 1:1:1 ratio directly at enrollment (exact allocation per arm may vary slightly depending on block randomization, approximately 27, 27, and 26 patients per arm, given block randomization). The primary analysis will be conducted on an intention-to-treat basis, including all randomized participants; a per-protocol analysis restricted to patients who complete the trial will be reported as a secondary analysis.

## 4 Discussion

As the global population ages, the socio-economic burden of dementia in developed countries, such as Italy has reached significant dimensions (ISTAT, 2020), necessitating urgent responses (CENSIS, 2024). Within this context, apathy emerges as one of the most prevalent and distressing neuropsychiatric symptoms in mild cognitive impairment, mild behavioral impairment, and dementia (Mortby et al., 2022). To date, apathy has largely remained excluded from the focus of available pharmacological intervention studies (NICE, 2018; O'Connor et al., 2019; Oliveira et al., 2021). The RAPHAel protocol is designed to address this gap by testing whether robotic interventions can deliver structured stimulation and social engagement directly in the home setting. Namely, our protocol aims to migrate specialized rehabilitation from institutional settings directly into the patients’ homes, potentially extending personalized care to a broader population.

Despite the growing interest in socially assistive robotics for individuals with cognitive impairment, robust evidence supporting the clinical effectiveness of home-based robotic interventions specifically targeting apathy remains limited. Previous studies have generally involved small samples, focused primarily on feasibility or usability, or evaluated assistive rather than rehabilitative robotic systems (Hung et al., 2021; Mancioppi et al., 2019; Moyle et al., 2019). Moreover, few randomized controlled trials have directly compared different robotic approaches with an active standard-of-care intervention. The RAPHAel trial has been specifically designed to address these gaps by evaluating two complementary robot-assisted rehabilitation strategies against occupational therapy within a randomized controlled framework, while simultaneously integrating clinical, behavioral, and technology-derived outcome measures.

A fundamental innovation of the RAPHAel project is its shift from purely “assistive” robotics toward a “rehabilitative” model. By leveraging the principles of neural plasticity and cognitive reserve (Stern, 2006; Valenzuela and Sachdev, 2006; Esiri and Chance, 2012), we hypothesize that targeted non-pharmacological interventions can stimulate capabilities even in the presence of neurodegeneration.

The RAPHAel project employs a three-arm randomized controlled design to evaluate two distinct home-based technological interventions as compared to the current gold standard of occupational therapy. The first experimental approach features a tele-operated system (RoboXcare) dedicated to structured cognitive stimulation. This intervention, remotely facilitated by a neuropsychologist, targets specific domains, such as attention, memory, and executive functions, through a validated protocol characterized by systematically increasing difficulty levels. On the other hand, the second arm utilizes Pepper, an autonomous humanoid robot designed for conversational engagement; by connecting to a dedicated CAIR server, the system facilitates personalized dialogues tailored to each patient’s interests and life history, providing a platform for spontaneous social interaction without restrictions on frequency or duration.

Furthermore, the implementation of these interventions in the ecological context of the patient’s home offers unique advantages. Not only does it make the treatment more realistic and generalizable to daily life, but it also provides crucial support to caregivers, who often face high levels of stress and reduced quality of life. Consistently with recent trends in digital therapeutics (Matamala-Gomez, et al., 2020; Moccia, et al., 2021), RAPHAel also aims to pioneer the development of “digital markers”. By collecting in-depth metrics, such as social grasping kinematics and conversational metrics, we expect to establish objective measures for monitoring behavioral symptoms and the efficacy of future treatments.

### 4.1 Anticipated results

The RAPAHel study has two primary expected outcomes. The first is to generate data useful for the development of digital biomarkers of behavioral disturbances in individuals with cognitive decline, with the purpose to facilitate the monitoring of the effectiveness of both pharmacological and non-pharmacological interventions. The second is to demonstrate the feasibility of robot-mediated interventions in the home setting for individuals with cognitive decline and related behavioral disturbances, yielding both direct benefits (for individuals with cognitive impairment or dementia) and indirect benefits (for caregivers/family members).

We anticipate that participants receiving the robot-assisted interventions will show improvements in apathy and social engagement relative to the baseline and that such improvement is comparable to the active occupational therapy control group, while also demonstrating good usability and acceptance of the proposed technologies, one of which (Pepper) has never been used at the patient’s home. We further expect that interaction metrics collected by the robotic platforms and kinematic parameters derived from the social grasping task will provide objective digital markers associated with behavioral changes and treatment response. These measures may contribute to the development of more personalized rehabilitation strategies for individuals with cognitive impairment.

Beyond the expected clinical benefits, the study is designed to evaluate the feasibility of implementing home-based robotic rehabilitation in real-world settings. Positive findings would support the integration of robotic technologies into routine clinical practice and provide evidence for scalable, home-based interventions that may reduce caregiver burden while increasing access to personalized rehabilitation.

While we acknowledge potential limitations, such as the pragmatic sample size common in robotic trials, we expect that the findings of this randomized controlled trial will clarify whether home-based robotic interventions are usable, safe, and potentially effective in reducing apathy-related behavior. In summary, the RAPHAel project will provide evidence on the feasibility, acceptability, and potential clinical value of home-based robotic interventions for apathy in individuals with cognitive decline, shaping the development of sustainable models for the healthcare systems of the future.

Ultimately, the RAPHAel trial is expected to provide an important step toward establishing evidence-based home-based robotic rehabilitation for apathy in cognitive impairment, while informing future multicenter trials and supporting the integration of personalized robotic technologies into routine dementia care.

## 5 Limitations

This study presents some intrinsic limitations, due to the implementation of innovative home-based robotic interventions. A first limitation concerns the unequal and non-standardized contact time across study arms. The occupational therapy control group follows the frequency defined by national care standards (Ministero della Salute, DPCM, 2017), the telerobot arm follows a fixed schedule of three 45-min sessions per week, while the Pepper robot arm allows for flexible, self-paced interactions with no upper limit. This design choice reflects the practical and technical characteristics of each modality rather than an attempt to equalize contact time, and it does not allow a complete separation between intervention-specific effects and non-specific effects related to attention and social contact, particularly in the comparison between the robotic arms and the active control group. Actual contact time in each arm will be recorded and may be used as a covariate in sensitivity analyses.

Furthermore, potential limitations include participant drop-out, variability in digital literacy among patients and caregivers, technical issues related to internet connectivity or device functioning, and differences in disease severity that could influence adherence to the intervention. To minimize these issues, all participants and caregivers will receive standardized training before the intervention, technical support will be available throughout the study, and the occupational therapist responsible for device installation will verify the correct functioning of the equipment. In addition, the pragmatic design of the trial and the relatively limited sample size should be considered when interpreting the findings. Nevertheless, the randomized controlled design and the inclusion of an active control group are expected to provide robust preliminary evidence regarding the feasibility, acceptability, and potential clinical effectiveness of robot-assisted interventions for apathy.

## Data Availability

The original contributions presented in the study are included in the article/supplementary material, further inquiries can be directed to the corresponding author.
